# Environmental Occurrence, Toxicity Concerns, and Degradation of Diazinon Using a Microbial System

**DOI:** 10.3389/fmicb.2021.717286

**Published:** 2021-11-01

**Authors:** Xiaozhen Wu, Jiayi Li, Zhe Zhou, Ziqiu Lin, Shimei Pang, Pankaj Bhatt, Sandhya Mishra, Shaohua Chen

**Affiliations:** ^1^State Key Laboratory for Conservation and Utilization of Subtropical Agro-bioresources, Guangdong Province Key Laboratory of Microbial Signals and Disease Control, Integrative Microbiology Research Centre, South China Agricultural University, Guangzhou, China; ^2^Guangdong Laboratory for Lingnan Modern Agriculture, Guangzhou, China

**Keywords:** diazinon, toxicity, abiotic degradation, microbial degradation, degradation pathways, catalytic mechanisms

## Abstract

Diazinon is an organophosphorus pesticide widely used to control cabbage insects, cotton aphids and underground pests. The continuous application of diazinon in agricultural activities has caused both ecological risk and biological hazards in the environment. Diazinon can be degraded via physical and chemical methods such as photocatalysis, adsorption and advanced oxidation. The microbial degradation of diazinon is found to be more effective than physicochemical methods for its complete clean-up from contaminated soil and water environments. The microbial strains belonging to *Ochrobactrum* sp., *Stenotrophomonas* sp., *Lactobacillus brevis*, *Serratia marcescens*, *Aspergillus niger*, *Rhodotorula glutinis*, and *Rhodotorula rubra* were found to be very promising for the ecofriendly removal of diazinon. The degradation pathways of diazinon and the fate of several metabolites were investigated. In addition, a variety of diazinon-degrading enzymes, such as hydrolase, acid phosphatase, laccase, cytochrome P450, and flavin monooxygenase were also discovered to play a crucial role in the biodegradation of diazinon. However, many unanswered questions still exist regarding the environmental fate and degradation mechanisms of this pesticide. The catalytic mechanisms responsible for enzymatic degradation remain unexplained, and ecotechnological techniques need to be applied to gain a comprehensive understanding of these issues. Hence, this review article provides in-depth information about the impact and toxicity of diazinon in living systems and discusses the developed ecotechnological remedial methods used for the effective biodegradation of diazinon in a contaminated environment.

## Introduction

**FIGURE 1:**
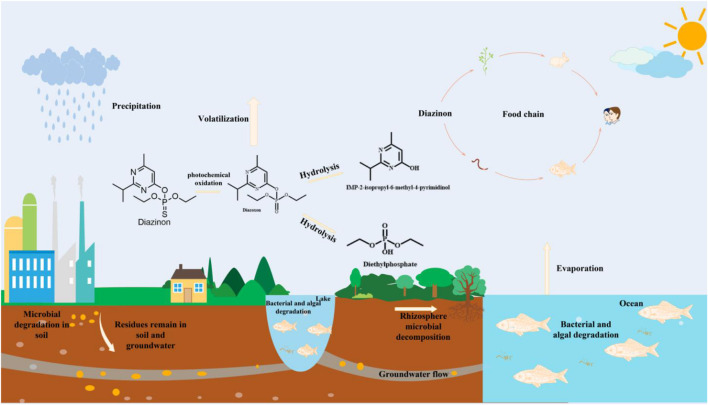
The fate and occurrence of diazinon in the environment.

With the rapid development of agriculture, organophosphorus pesticides (OPs) are characterized by specificity, broad spectrum applicability, and high efficiency. They play a prominent role in the control of agricultural pests and diseases. Since 1960, OPs have occupied the highest market share of pesticides (19% of the world market) (Villiot et al., 2018). Diazinon (*O*,*O*-diethyl-*O*-[6-methyl-2-(1-methyl-ethyl)-4-pyrimidine] thiophosphate) is a broad-spectrum, highly effective, medium–low toxicity organophosphate insecticide. It is one of the most commonly detected OPs in groundwater, drinking water and surface water, which is an especially serious problem (Cao et al., 2018; Glinski et al., 2018). Environmental residues of diazinon can cause harm to nontarget organisms through the air, water, soil, and food chain (Figure 1).

During the application process, only 10% of the pesticides used can reach the target organisms, and the remaining 90% is distributed in the environment. High concentrations of diazinon can be easily detected in the rivers of the United States, Italy, Spain, China and other countries (Hajirezaee et al., 2017; Al-Otaibi et al., 2019; Mena et al., 2020). Diazinon has a thiophosphate backbone, which is metabolized by cytochrome P450 to form the OP-oxon form, which inhibits acetylcholinesterase (AChE), causes nerve tissue failure and kills insects. After accidental exposure to diazinon, fish, shrimp, shellfish and human children can develop neurological and developmental disorders, so they are restricted by the U.S. Environmental Protection Agency (Yen et al., 2011; Montuori et al., 2016; Sharma et al., 2019). Clearly, there is an urgent need to remove residual diazinon from the environment.

A high number of researchers have performed research studies around this topic, and the previously established degradation methods can be divided into abiotic degradation and microbial degradation (Kumar et al., 2018; Mulla et al., 2018; Baharum et al., 2020; Sikakwe et al., 2020). Diazinon can be further degraded through continuous optimization of physical and chemical conditions. Previous studies showed that copper-doped ZnO nanorods could overcome the disadvantages of ZnO nanoparticles as nanocatalysts and could perform photocatalytic degradation of organophosphorus pesticides, such as diazinon, with a degradation efficiency of 96.97%, which was more valuable than the UV/ZnO process (Shirzad-Siboni et al., 2017). Alalm et al. (2015) used a combination technique. In the first stage, a solar heterogeneous TiO_2_ photocatalyst was used, powdered activated carbon (PAC) was selected for adsorption, and nearly 100% of diazinon was removed. Further study found that there are three main byproducts, namely, diazoxon 7-methyl-3-octyne, 2-isopropyl-6-methyl-4pyrimidinol and diethyl phosphonate (Toolabi et al., 2018). However, abiotic degradation still has some disadvantages, such as incomplete degradation, high production cost, and complex operation (Arora, 2020; Saleh et al., 2020). Thus, it is very important to develop cleaner, cheaper and easier removal technologies.

Microbial degradation of pesticides has the characteristics of high efficiency, low cost, environmental protection, and sustainability, which has attracted the attention of researchers (Mishra et al., 2020; Li et al., 2021; Lin et al., 2021). Many previous studies have shown that biodegradation of diazinon is a promising approach for the remediation of diazinon-contaminated environments. These microorganisms include *Stenotrophomonas*, *Bacillus serrata*, *Burkholderia*, *Streptomyce*s, and *Aspergillus niger*, which are highly effective in the removal of diazinon when provided appropriate growth conditions (Cycoń et al., 2009; Góngora-Echeverría et al., 2020; Hamad, 2020). The reason why these microorganisms can effectively degrade diazinon lies in the various enzymes contained in their bodies. These enzymes have high enzyme activity and a variety of hydrolysis and oxidation functions, which can turn the pollutants into short chain products with low toxicity. However, the pH, temperature, and low stability properties of these enzymes limit their use in industrial applications (Bhatt et al., 2020b; Mishra et al., 2021). At present, people use the immobilization technology of enzymes to improve the thermal stability, reduce the inhibition of the product, and overcome the common difficulty of solubility. However, researchers do not have the complete system needed to define the effective degrading enzymes contained in microorganisms. This increases the difficulty of subsequent studies on the degradation of diazinon. A review of relatively complete and clear degradation pathways and construction of related degradation enzymes is of great reference value.

This article will discuss both the role in and toxicity of diazinon in life systems and explain the application of microbial strains to the degradation of diazinon. In addition, the mechanisms and kinetics of local microbial strains were compared, and they were found to be effective for the degradation of diazinon. We will focus on the degradation pathways and catalytic mechanisms of diazinon to better understand how microorganisms can enhance the degradation of diazinon, thereby working towards rectifying its dispersion in the natural environment.

## Toxicity of Diazinon

Diazinon is widely used in the control of various insects and can be used as an insecticide in agricultural production systems. The related properties of diazinon are shown in Table 1. In the field of veterinary medicine (Mitra and Maitra, 2018), diazinon is often used as an acaricide and as an insect repellent sprayed on livestock and poultry. At the same time, it was also categorized as a moderately hazardous pollutant of class II by the World Health Organization (Pirsaheb et al., 2014; Jonidi-Jafari et al., 2015). In the United States, the phasing-out of diazinon for indoor and outdoor use began in 2002. Diazinon is highly effective in pest control and is widely used in fruits, vegetables, nuts, and ornamental products, and up to 100 tons can be used per year (Shrestha et al., 2018). In Iran and other Middle Eastern countries, it is used in grape cultivation to good effect (Bakırcı et al., 2014; Pirsaheb et al., 2017; Philippe et al., 2021). However, continuous use of diazinon causes it to accumulate in the environment and damage the health of nontarget organisms by entering the food chain.

**TABLE 1:** Physical and chemical properties and structure of diazinon (Malakootian et al., 2020).

Description	Properties
Molecular structure	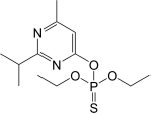
IUPAC name	*O*,*O*-Diethyl *O*-2-isopropyl-6-methyl-4-pyrimidinyl phosphorothioate
Molecular formula	C_12_H_21_N_2_O_3_PS
Molar mass (g/mol)	304.3
Density	1.116–1.118 (20°C)
Toxicity	LD_50_ (mg/kg)
Octanol–water Partition coefficient, logK_*ow*_	3.81
Solubility in water	40 mg/L at 25°C
Dissociation constant (pKa) at 25°C	2.6

Because of the trend towards large-scale usage of pesticides, the pollution sources of pesticides are not only limited to the intensive use of pesticides in urban areas but can also be linked to the chemical industry and farmland (Liu et al., 2015; Wee et al., 2016; Zainuddin et al., 2020). Pesticides and their residues (metabolites) can condense into rain through surface runoff, soil leaching and transpiration, be deposited on the surface or in tissues of plants, enter drinking water and groundwater, and eventually reach nontarget organisms, including humans (Palma et al., 2014; Chaza et al., 2018; Sumon et al., 2018; Villiot et al., 2018; Triassi et al., 2019). The levels of diazinon in several water sources have been found to exceed the standard, and the treatment processes used in diazinon agrochemical plants and sewage treatment plants still cannot fully degrade pesticide residues (Fadaei et al., 2012; Arellano-Aguilar et al., 2017; Hamad, 2020). Coming into contact with diazinon triggers the phosphorylation of cholinesterase *in vivo* (Glavan et al., 2018; Díaz-Resendiz et al., 2019). Acetylcholinesterase is inactivated and toxic. A large number of pesticides in the environment continue to accumulate, not only inhibiting insect acetylcholinesterase activity but also interfering with the nervous system of various organisms, causing neurotoxicity (Hajirezaee et al., 2017; Glavan et al., 2018; Mena et al., 2020). Čolović et al. (2015) also found that diazinon metabolites were nontoxic, but their stimulation by superoxide dismutase was up to 30%, and a high concentration of diazinon and its metabolites had a significant effect on lactate dehydrogenase activity.

Diazinon is also very harmful to aquatic organisms, especially local shellfish species in aquatic ecosystems (such as *Digueti* and *Daphnia*); under the stress of diazinon, its potential acute toxicity gradually reached a very high level (Arias-Andrés et al., 2018; Chen et al., 2018). In addition, 4.5 mg/L diazinon inhibited acetylcholinesterase in amphibian embryos, leading to endothelial cell changes and body length shortening and eventually leading to biological deformities (Aronzon et al., 2014). Velki et al. (2017) used an *in vivo* model of zebrafish to evaluate the effects of the commonly used insecticide diazinon on the early life stages of zebrafish, and the results showed that diazinon had influence on enzyme reactions and gene expression changes. Toledo-Ibarra et al. (2016) evaluated lipid and protein oxidative damage in Nile tilapia exposed to diazinon and found that proteins in the gills and liver tissues were more easily oxidized by diazinyl than lipids. In a recent study, Hajirezaee et al. (2017) reported, for the first time, the adverse effects of the exposure of Persian sturgeon larvae to diazinon on their seawater adaptation. Intestinal microorganisms in mice decompose organophosphates, including diazinon, into gluconeogenic substrates, which interfere with the normal activities of intestinal microorganisms, leading to glucose regeneration and glucose intolerance, thereby increasing the incidence of diabetes (Gao et al., 2017; Velmurugan et al., 2017).

Pesticides can affect host health in many ways, such as altering the composition of gut microbes and their metabolites. The introduction of diazinon into mice significantly reduced the bacterial population of the *Lachnospiraceae* family, which is involved in the production of short-chain fatty acids, caused bile acid disorder (Gao et al., 2017; Adamovsky et al., 2018), and destroyed intestinal mucosa and intestinal cells (Groh, 2017; Gillois et al., 2018). With the destruction of the intestinal flora balance and the enhancement of intestinal permeability, more lipopolysaccharides (LPS) are introduced into the body, ultimately triggering low-level inflammation (Ghetti, 2018; Liang et al., 2019). Large amounts of organophosphorus pesticides can inhibit acetylcholinesterase (AChE) in the central and peripheral nervous systems and promote an increase in acetylcholine, which can lead to nausea, headache, psychosis, depression, memory loss, chronic fatigue syndrome, and respiratory problems (Sultatos, 2006).

This series of environmental questions has constantly perplexed modern human beings. Finding a treatment technology with low price, complete degradation, and no secondary pollution through sustainable development is particularly important.

## Abiotic Degradation of Diazinon

### Physical and Chemical Degradation of Diazinon

With increasing interest in diazinon, many studies have been carried out regarding its degradation. Physicochemical degradation is one of the most widely used methods, including photocatalyst treatment, advanced oxidation treatment, biological treatment membrane filtration, and ion exchange treatment (Hassan et al., 2017; Kumar et al., 2018; Pordel et al., 2019). The physical and chemical degradation methods of diazinon are shown in Table 2. Physical adsorption and chemical degradation are the main techniques used for pesticide degradation. A variety of adsorbents have been developed and used, and the optimization conditions of photocatalysts have also been archived. It has been proven that these methods are effective, but the use cost is relatively high (Jonidi-Jafari et al., 2015). In this type of experiment using TiO_2_, TiO_2_ particles cannot be separated from a solution after treatment (Baharum et al., 2020). The cost of the UV/ZnO photocatalysis process is high, and the links to serious environmental problems include the environmental hazards of mercury vapor lamps, including the high toxicity of mercury and the short lifespan of the lamps themselves (Hossaini et al., 2017). Considering the solubility and persistence of diazinon, an appropriate technology can be used to remove diazinon from water systems.

**TABLE 2:** Physical and chemical methods for diazinon degradation.

Processing methods	Reaction conditions	Comments	References
Fe-TiO_2_/Bent-Fe photocatalysis	0.5 g/L of catalyst Visible light (36-W compact bulb) pH = 5.6	58.3% of diazinon (25 mg/L) was degraded within 6 h	Phuong et al., 2019
WO_3_ photocatalysis	0.5 g/L of catalyst UV light (125- W medium-pressure UVC lamp) pH = 3	99.88% of diazinon (20 mg/L) was degraded within 2 h	Mohagheghian et al., 2016
Fe-TiO_2_ photocatalysis	0.1 g/L of catalyst UV light (125-W medium-pressure UVC lamp) pH = 7	98.53% of diazinon (50 mg/L) was degraded within 2 h	Dehghani et al., 2019
MgO photocatalysis	0.1 g/L of catalyst UV light (5 lamps) pH = 7	99.46% of diazinon (5 mg/L) was degraded within 2 h	Ahmadifard et al., 2019
Iron doped TiO_2_ photocatalysis	0.4 g/L of catalyst UV light (15-W low pressure UV lamp) pH = 5.5	76% of diazinon (30 mg/L) was degraded within 100 min	Tabasideh et al., 2017
Cu-doped ZnO nanorods	0.2 g/L of catalyst Gasoxygen gas = 2 L/min pH = 7	96.97% of diazinon (50 mg/L) was degraded within 2 h	Shirzad-Siboni et al., 2017
WO_3_ nanostructures	WO_3_ nanostructures: sulfuric acid (H_2_SO_4_) 1.5M, nitric acid (HNO_3_) 1.5M, methanesulfonic acid (CH_4_O_3_S) 1.5M UV light (500W xenon lamp)	90% of diazinon (20 mg/L) was degraded within 24 h	Roselló-Márquez et al., 2021
WO_3_-doped ZnO photocatalysis	10 mg/cm^2^ of catalyst UV light (6-W low pressure lamp) pH = 7	89% of diazinon (20 mg/L) was degraded within 2 h	Maleki et al., 2020
WO_3_-doped ZnO photocatalysis	10 mg/cm^2^ of catalyst Sunlight pH = 7	83% of diazinon (20 mg/L) was degraded within 2 h	Maleki et al., 2020
Chemically modified phosphoric acid adsorption	5.0 g/L of adsorbent pH = 7	98.96% of diazinon (1.0 mg/L) was degraded within 2 h	Baharum et al., 2020
Adsorption of multi-walled carbon nanotubes	0.1 g/L of adsorbent pH = 4	99.1% of diazinon (0.3 mg/L) was degraded within 15 min	Dehghani et al., 2019

Considering the high content of diazinon in water, there are many methods to improve the removal rate of diazinon by optimizing the characteristics of adsorbents. The vast majority of researchers use a mixture of biochar, activated carbon, minerals, clays, and certain metal–organic frameworks (MOFs) as adsorbents to remove pesticide residues (Abdelhameed et al., 2017; Derylo-Marczewska et al., 2017; Abdelhameed et al., 2019; Durán et al., 2019; Emam and Shaheen, 2019; Baharum et al., 2020). Biochar is prepared from agricultural and forestry production wastes such as raw biomass materials (Ponnam et al., 2020). It has the advantages of loose and porous features, a large specific surface area and high surface energy, which can greatly improve the removal efficiency (Ding et al., 2017). It is one of the adsorbents for pesticide removal (Baharum et al., 2020; Okoya et al., 2020). In addition, it can effectively remove organic pollutants in water, such as dyes and drug compounds (Tran et al., 2020; Wu et al., 2020).

Previous research has shown that organic materials play an important role in the use of agricultural waste to remove pesticides. Using waste coconut biomass for modification, carbonized blonde shell biochar (BC1), activated blonde shell biochar (BC2), chemically modified phosphoric acid (BC3), and sodium hydroxide blonde shell biochar (BC4) were prepared as adsorbents for the removal of diazinon (Baharum et al., 2020). When the pH was 7, the dosage was 5.0 g/L, and the adsorbent was BC3, the removal rate of diazine reached 98.96%. Similarly, a large amount of agricultural waste can be used for pesticide removal, such as corn stalks, rice stalks, discarded orange peel, almonds, wood derivatives, birch and Norwegian spruce, bamboo flakes, and even poultry dung, all of which have achieved good results (Liu et al., 2015; Cederlund et al., 2016; Mandal et al., 2017; Suo et al., 2019; Abdelhamid et al., 2020).

Due to the transfer of diazinon from the liquid phase to the solid phase in the adsorption process, secondary contamination can easily occur, which can increase the treatment cost. Photocatalysis is a pesticide removal technology exhibiting complete oxidation, a simple product structure, high efficiency, and a low-cost catalyst, as well as reducing secondary pollution and simultaneously destroying organic pollutants. Mirmasoomi et al. (2017) reported a maximum photocatalytic degradation rate of diazinon of up to 95.07% using a TiO_2_/Fe_2_O_3_ nanocomposite as a catalyst under visible light conditions. Mohagheghian et al. (2016) investigated the photodegradation of diazinon with nano-WO_3_ powder as a catalyst under ultraviolet light irradiation, and the removal efficiency was unexpectedly much higher. Phuong et al. (2019) showed that the initial concentration was set at 25 mg/L and the degradation rate was 58.3 in Fe-TiO_2_/Bent-Fe photocatalysis. Nakaoka et al. (2010) reported that the removal rate of diazinon was approximately 88% after 30 h of treatment with platinized TiO_2_ as the catalyst using UV irradiation. Maleki et al. (2020) studied WO_3_-doped ZnO photocatalysis, in which the mineralization rate of 20 mg/L diazinon under UV irradiation reached 89%. The ozone degradation of diazinon is performed using nanometal oxides as catalysts, and it generates a variety of active free radicals, which accelerate the additional reaction of hydroxyl radicals and the oxidation of the phosphate group, which is, in turn, conducive to the removal of diazinon (Malakootian et al., 2020). However, a change in external conditions will cause a reduction in free radicals and stimulate the reaction competition of hydroxyl radicals.

Recently, researchers not only continued to optimize the treatment of diazinon but also carried out in-depth studies on its degradation mechanism, which provides a greater scientific basis for our review.

### Physico-Chemical Degradation Mechanism of Diazinon

With the increasing amount of attention being paid to diazinon, abiotic hydrolysis has become one of its main degradation pathways. Under acidic or alkaline conditions, the nitrogen and phosphorus groups in diazinon are activated by pyrimidine protons, which initiate nucleophilic attacks, break the phosphorus and oxygen bonds, and eventually cause rapid hydrolysis. At higher pH values, the excitor of the nucleophilic attack may be one of the sulfur, benzene, nitrooxy, or pyrimidine protons. The hydrolysates in these cases are the less toxic 2-isopropyl-6-methyl-4-quill (IMP) and diethyldithiophosphoric acid. Consequently, several treatment methods for the degradation of diazinon have been suggested, such as chlorination, ultrasonic irradiation, Fenton’s reagent, photoFenton, UV/O_3_, UV/H_2_O_2_, UV/ZnO, UV/TiO_2_, UV/ZnO/TiO_2_, and solar/advanced oxidation processes (AOPs), each of which provide efficient degradation of diazinon (Li et al., 2015; Alvarez-Corena et al., 2016; Soto-Vázquez et al., 2016; Hossaini et al., 2017; Shirzad-Siboni et al., 2017; Tabasideh et al., 2017; Ayoubi-Feiz et al., 2018). In AOPs, UV/H_2_O_2_ is considered to be an effective method for the treatment of organophosphorus pesticide and other micro-organic pollutants (Shemer and Linden, 2006). In the presence of ultraviolet light, the hydrophobic part of natural organic matter (NOM) in water was destroyed, and hydrophilic or polar degradants were generated (Wols and Hofman-Caris, 2012), while haloacetic acid formation increased upon chlorination, which was conducive to the degradation of diazino-organic pesticides. In addition, compared with direct UV photolysis, the UV/H_2_O_2_ combined process can mineralize diazinon to a higher degree (Sarathy and Mohseni, 2010).

Sajjadi et al. (2019) used a Fe_3_O_4_@MOF-2 nanocomposite (MOF: metal–organic framework) as a catalyst to excite persulfate (PS) under ultrasonic radiation (US) and acidic conditions, which increases the photocatalytic activity and enhances the generation of hydroxyl sulfate radicals in an aqueous solution. Combined with ultrasonic acoustic cavitation, hydroxy easily reacts with H_2_O and O_2_ to generate H_2_O and H_2_O_2,_ thus promoting the decomposition of organic pollutants (Sajjadi et al., 2019). Similarly, under UV and US irradiation, the absorption of photons on the surface of the *N*-doped TiO_2_ catalyst increases, the availability of active sites on the surface of TiO_2_ increases, and photoactivated light permeates into the suspension, having a positive effect on the ACo photocatalytic degradation of diazinon (Ayoubi-Feiz et al., 2018). In the UV/Cu-doped ZnO process system, the shortcomings of ZnO nanoparticles as nanocatalysts are overcome. The dissolved oxygen is transformed into superoxide anion (O_2_⋅^–^), and then, the excited electrons in the photocatalyst react with the electrons in the reaction system, inhibiting the recombination of positive holes and electrons. The degradation efficiency is as high as 96.97%, which is more valuable than the UV/ZnO process (Jonidi-Jafari et al., 2015). Hossaini et al. (2017) reported that the cns-ZnO/LED process could reduce the accumulation of diazinon in the environment as the system’s specific surface area increased by approximately 30%, meaning that LED radiation can activate more reaction sites. In addition, Liu et al. (2009) found that acid sites were provided in the structure where the catalyst existed, preventing the generation of electron–hole compounds and improving the removal efficiency of organic pollutants. At present, active photocatalysts for photocatalytic degradation of organic and inorganic pollutants are limited to certain metals and nonmetallic substances, such as titanium, tungsten oxide, zinc oxide, iron oxide, cadmium sulfide and zinc sulfide, which are gradually introduced (Daneshvar et al., 2007; Sajjad et al., 2018; Khoiriah et al., 2020; Maleki et al., 2020). The ZnO@SiO2@Fe3O4/PMS/UV system is beneficial to the decomposition of oxidizing agents, and a variety of reactive oxidizing species (H⋅, HO⋅, O_2_⋅^–^, SO_4_⋅^–^) are involved in the degradation of diazinon. H⋅ and HO⋅ species play a leading role in diazinon degradation (Daneshvar et al., 2007; Rezaei et al., 2019; Maleki et al., 2020). A number of studies have reported that ozone is a strong oxidant with a redox potential of 2.07 V, which can be combined with UV, H_2_O_2_ and other processes to destroy ozone and generate more HO⋅ radical oxidation of diazinon to form short-chain compounds (Ayoubi-Feiz et al., 2019; El Hassani et al., 2019; Malakootian et al., 2020).

These combined technologies have been tested and successfully applied to the treatment of diazinon-contaminated sites. Due to their high efficiency, high safety index and positive environmental benefits, the degradation products and pathways of diazinon have been explored (Badawy et al., 2006; Alvarez-Corena et al., 2016; Soto-Vázquez et al., 2016; Orge et al., 2017). The formation of intermediate products for the degradation of diazinon has been widely reported. Arief et al. (2015) showed that the intermediate diazoxon could be rapidly hydrolyzed under both acidic and alkaline conditions, but it was unstable under ultraviolet light conditions and could be oxidized to form diazoxon, but that its toxicity was stronger than that of the parent compound (Okoli et al., 2017). Li et al. (2015) reported that diazinon is hydrolyzed to form ((Z)-3-((E)-1-hydroxy-2-methylpropylidene)amino)but-2-enimidic acid (IMP) in a UV/H_2_O_2_ combination by means of cleavage of the P-O bond (pyrimidine ring). With further control of the system of the environmental conditions, hydrogen base and oxhydryl participates in the additional reaction of IMP, the formation structure is relatively simple, 2-isopropyl-6–4-ol methylhexahy- dropyrimidin-, (((Z)-3-(Z)-1- hydroxy-2-methylpropylidene) amino) but-2-enoic acid, 6-methylhexahydropyrimidine-2,4-diol, (Z)-3-(((Z)-1-amino-2-methylpropylidene) amino) but-2-enoic acid. Rezaei et al. (2019) also reported another pathway of diazinon, in which hydroxyl diazinon and 2-hydroxyl diazinon can be generated by hydroxylating primary and tertiary carbon atoms of the propyl group, and then the hydroxyl radical acts on the O functional group and diazinon is hydrolyzed to produce diethyl phosphate and IMP.

**FIGURE 2:**
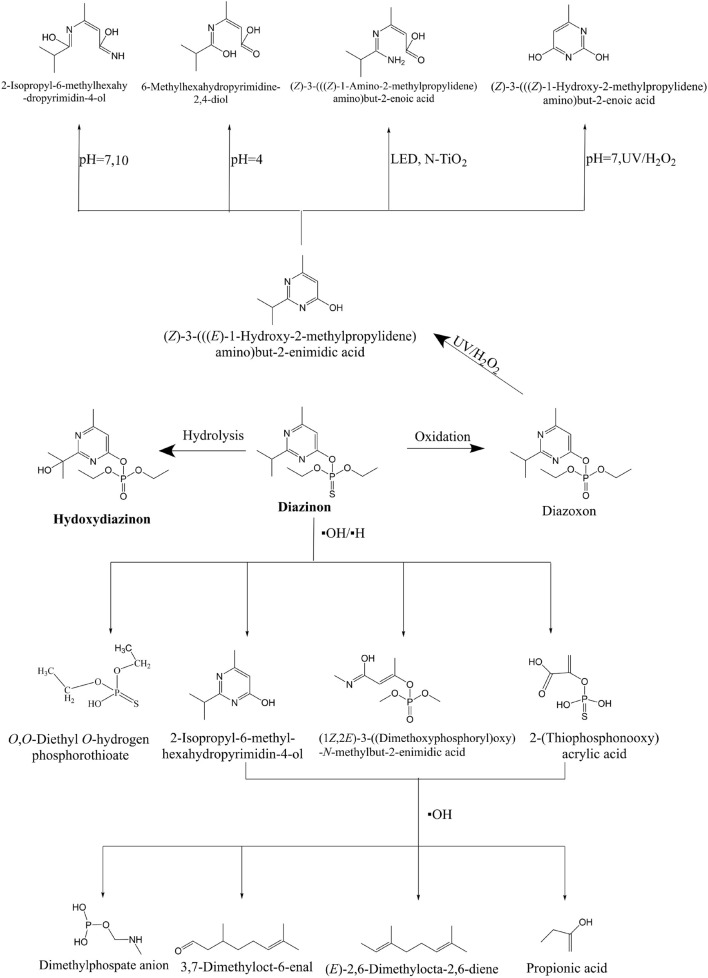
Proposed physico-chemical degradation pathways of diazinon (Arief et al., 2015; Čolović et al., 2015; Malakootian et al., 2020). The thick black arrows point to the major degradation products.

In general, the degradation pathway of diazinon is mainly the substitution of sulfur by oxygen in the P = S bond, hydroxylated oxidation of the C-N bond, and cleavage of the C-O bond. Previously, the main degradation products of diazinon were hydroxydiazoxon and IMP. The specific degradation path of diazinon is shown in Figure 2.

## Microbial Degradation of Diazinon

Although the above abiotic degradation methods can achieve a high removal efficiency, there are still some problems, such as the high cost of equipment, uncertainty regarding intermediate products and incomplete mineralization (Chen et al., 2012; Zhan et al., 2018b; Bhatt et al., 2021a). Therefore, microbial degradation technology is favored because of the distinct advantages of being low-cost, safe, and effective, providing complete degradation and producing no secondary pollution (Liu et al., 2007; Qiu et al., 2018; Roman et al., 2019).

Bacteria, fungi, actinomycetes, and algae that can remove diazinon were obtained by enrichment cultures (Cycoń et al., 2009; Pourbabaee et al., 2018; Hamad, 2020). Diazinon-specific degrading microorganisms are shown in Table 3. Researchers have used enrichment techniques to search for microorganisms that can be used to mineralize diazinon to reduce the concentrations of diazinon in soil agricultural wastewater discharge systems, seawater systems, and heavy industry (Briceño et al., 2015; Wang and Liu, 2016). However, only a small number of microorganisms have been isolated and identified.

**TABLE 3:** Indigenous microbial strains involved in diazinon degradation.

Microbial strains	Strain type	Source	Comments	References
*Lactobacillus brevis*	Bacterium	Center of Lactic Acid Bacteria in Key Laboratory of Dairy Science, Northeast Agricultural University, China	About 52% of diazinon (0.6 mg/L) was degraded within 24 h	Zhang et al., 2014
*Stenotrophomonas* sp.	Bacterium	Industrial sludge (China)	Nearly 100% of diazinon (50 mg/L) was degraded within 24 h	Deng et al., 2015
*Ochrobactrum* sp.	Bacterium	Sludge from wastewater (China)		
*Serratia marcescens* DI101	Bacterium	Agricultural soil (Saudi Arabia)	Diazinon (50 mg/L) was completely degraded within 11 days	Abo-Amer, 2011
*Leuconostoc mesenteroides*, *L. brevis*, *L. plantarum*, *L.* sakei	Bacterium	Kimchi during fermentation (Korea)	About 74% of diazinon (100 mg/L) was degraded within 12 days at pH = 3.65-3.69	Cho et al., 2009
*Serratia liquefaciens*, *S. marcescens*, *Pseudomonas* sp.	Bacterium	Agricultural soil (Poland)	(1) About 80-92% of diazinon (50 mg/L) was degraded within 14 days (2) Utilizes diazinon as the sole carbon source (3) Adding other carbon sources (glucose) increases the decomposition rate	Cycoń et al., 2009
*Arthrobacter* sp., *Mycobacterium* sp.	Bacterium	Petroleum-contaminated soil (Hilo, Hawaii, United States)	These strains can utilize diazinon as growth substrate and transform diazinon.	Seo et al., 2007
*Streptomyces* sp. AC1-6., *Streptomyces* sp. ISP4	Bacterium	Agricultural soil (Chile)	(1) About 40-50% of diazinon (25 mg/L) was degraded within 24 h (2) About 70-90% of diazinon (50 mg/L) was degraded within 96 h	Briceño et al., 2015
*Flavobacterium* sp. ATCC 27551	Bacterium	Agricultural soil (United States)	About 95% of diazinon (50 mg/L) was degraded within 24 h	Mulbry and Karns, 1989
*Ralstonia* sp. DI-3	Bacterium	Agricultural soil (Huaibei, China)	(1) Diazinon (100 mg/L) was completely degraded within 60 days (2) Utilizes diazinon as the sole carbon source	Wang and Liu, 2016
*Stenotrophomonas maltophilia*	Bacterium	Paddy soils (Mazandaran, Iran)	(1) Diazinon is the main carbon source (50 μg/mL) (2) About 90% of diazinon was degraded within 15 days	Pourbabaee et al., 2018
*Bacillus amyloliquefaciens* YP6	Bacterium	Rhizosphere of Lolium perenne (Guizhou, China)	Increases soluble phosphorus, produces indole-3-acetic acid (IAA) and iron carriers	Meng et al., 2019
*Pseudomonas citronellolis* strain ADA-23B	Bacterium	Soil-straw; 1:1, *v*/*v* (Mexico)	About 40% of diazinon (50 mg/L) was degraded within 16 h	Góngora-Echeverría et al., 2020
*Bacterial endophytes* in rice plant (*Oryzia sativa* L.)	Bacterium	Rasht, Iran	(1) Diazinon (20 mg/L) is the sole carbon source (2) About 3.79-58.52% of initial dose was degraded within 14 days	Nasrollahi et al., 2020
*Pseudomonas putida* D3	Bacterium	Southeastern Iran	About 91% of diazinon was degraded (40 mg/L) within 21 days	Hassanshahian, 2016
*Pseudomonas peli*, *Burkholderia caryophylli*, and *Brevundimonas diminuta*	Bacterium	Soil sample	Diazinon (20 mg/L) was completely degraded within 18 days	Mahiudddin et al., 2014
*Alcaligenes faecalis* DSP3	Bacterium	Chemical factory, China	About 90% of diazinon (100 μg/mL) was degraded within 10 days	Yang et al., 2005
*Bacterium Enterobacter* B-14	Bacterium	Australian soil	Diazinon (25 μg/mL) was completely degraded within 2 days	Singh et al., 2004
*Aspergillus niger* MK640786	Fungus	Lake Burullus	About 82% of diazinon (1.25 mg/L) was degraded after 7 days (2) Optimal conditions for metabolism are pH = 5, 30°C	Hamad, 2020
*Rhodotorula glutinis* and *Rhodotorula rubra*	Fungus	Tomato plants	(1) During the same period, the initial concentration of diazinon was reduced by 88% when *R. glutinis* was added compared with the control (2) During the same period, the initial concentration of diazinon was reduced by 88% when *R. rubra* was added compared with the control	Bempelou et al., 2013
*Saccharomyces cerevisiae*	Fungus	Tehran, Iran	About 96% of diazinon (2.5 mg/L) was degraded after 22.75 h	Ehrampoush et al., 2017

These toxic chemicals can be transformed/degraded by bacteria and fungi to form microtoxic or nontoxic small molecules (Dar et al., 2019; Huang et al., 2020; Zhang et al., 2021). Bacteria have been widely used in the bioremediation of pesticides because of their strong biochemical behavior, multiadaptability, and reproductive ability (Dzionek et al., 2016; Cycoń et al., 2017; Lin et al., 2020). Under normal circumstances, a single strain can achieve complete degradation of diazinon (Cycoń et al., 2009). *Ralstonia* sp. DI-3 is a highly efficient diazinon-degrading bacterium isolated from agricultural soil. It can completely degrade diazinon at an initial concentration of 100 mg/L after just 60 h of liquid culture (Wang and Liu, 2016). This result is similar to the report of Abo-Amer (2012). When a small amount of glucose is added exogenously, it can promote the biodegradation of diazinon as a helper substrate (Cycoń et al., 2009). It has been shown that *Serratia liquifera*, *Serratia marcescens*, and *Pseudomonas* can use diazinon as the only carbon source in a mineral salt medium (MSM) containing 50 mg/L diazinon. These strains were able to degrade 80–92% of pesticides within 14 days (Cycoń et al., 2009). *Bacillus amyloliquefaciens* YP6, a growth-promoting rhizosphere bacterium, has been reported to effectively degrade organophosphorus pesticides (OPS). Seo et al. (2007) reported that *Arthrobacter* and *Mycobacterium* isolated from petroleum-contaminated soils were very effective in increasing the rate of diazinon mineralization. It was also found that *Arthrobacter* could not only hydrolyze diazinon but also remove other organophosphorus pesticides (such as chlorpyrifos, acetophosphorus, isophos and parathion).

For *Serratia marcescens* DI101 in a minimal salt medium, 50 mg/L diazinon was completely degraded in a period of 11 days compared to *Stenotrophomonas* sp. G1 strains, which degraded 50 mg/L diazinon within 24 h (Deng et al., 2015). Furthermore, it is worth noting that *S. marcescens* is key in the generation of diethyl phosphate, with organic phosphorus sulfur as a source of carbon bonds and phosphorus, such as chlorpyrifos coumaric, phosphorus parathion, and different nitrogen and phosphorus compounds in this category (Abo-Amer, 2011). The specificity of *Stenotrophomonas* sp. G1 metabolism is also related to pesticide structure, which is capable of degrading triphosphate organophosphorus pesticides, such as phoxim methyl p-p-parathion methyl p-p-parathion, while the degradation of propiom bromophos and triazophos is relatively slow. Current studies have shown that various bacterial genera, such as *Stenomonas*, *Serratia*, *Burkholderia*, *Rodanobacteria*, *Reisella*, and *Pseudomonas*, all use diazinon as the only carbon source, which improves the reaction rate and promotes the degradation of diazinon. In addition, bacteria *B. altitudinis* DB26-R and *B. subtilis* subsp., isolated from various plant tissues (endophytic bacteria), have great potential to degrade various compounds (Afzal et al., 2014). They have high degradability potential for diazinon. In recent years and fungi have also been used in the degradation of OPs. Ramadevi et al. (2012) reported that they have broad-spectrum pesticide degradation characteristics, as well as biological safety, economic feasibility and highly efficient degradation activity tolerance and are widely used in the pesticide bioremediation of contaminated soil water systems; they can even grow in contaminated soil water systems with a chlorpyrifos concentration of 700 mg. Optimized by response surface methodology, *Aspergillus niger* MK640786 effectively reduced diazinon and achieved a degradation rate of 82% under incubation conditions of 30°C, an initial concentration of 25 mg/L, a pH value of 5 and an incubation time of 7 days (Chen et al., 2012; Carranza et al., 2016). *Aspergillus* had different degradation efficiencies of diazinon under different environmental conditions. Culturing in 30°C liquid medium for 5 days was not conducive to the degradation of diazinon, and the degradation rate was as low as 46%.

Debasmita and Rajasimman (2013) found that if *Aspergillus* were incubated for 14 days, the hydrolysis of diazinon could reach 90.02%. On the other hand, fungi, such as *Anisoplia bassiana*, could degrade 72% of chlorpyrifos within 132 h (Shah et al., 2017). The degradation rate was only 35.3% when cultured in medium containing diazinon for 4 days (Fareed et al., 2017). Ehrampoush et al. (2017) found that *Saccharomyces cerevisiae* could use diazinon (initial concentration of 1000 mg/L) as its carbon source. Diazinon was successfully degraded by *S. cerevisiae* within 0.5 h by 85.23%. The degradation rate of carmoisine dye was 96%.

In a pure culture of *Streptomyces* with a diazinon concentration of 50 mg/L, only 32% degradation was found, likely because its toxicity is greater than the original byproduct of degradation compounds. As a result, people began to use microbial populations of mixed culture alone or with other populations of common culture techniques in order to avoid the degradation process of the accumulation of toxic compounds, and the degradation effect was better than that of pure culture (Fuentes et al., 2011). When *Streptomyces* strains AC5, AC9, GA11, and ISP13 were used in a mixed culture (SMC), the degradation rate of diazinon reached the maximum (62%). Briceño et al. (2016) also investigated the removal effect of *Streptomyces* mixed cultures in 100 chlorpyrifos (CP) + diazinon (DZ)-contaminated liquid media. This will hopefully be an alternative approach to removing DZ from the environment. This approach uses an inorganic salt medium in which the enrichment cycle is run multiple times and diazinon isolated from paddy soil is mixed with degrading bacteria, consisting of species from *Burkholderia*, *Achromobacter*, *Hyphomicrobium*, *Rhodanobacter*, and so on. Within cultures of 16.81 and 19.60 days, pesticide degradation achieved favorable results, and the removal rate reached 90%. There are many similar situations reported. In the microbial remediation experiment, it was found that when the mixture of strains degraded diazinon (Briceño et al., 2016), the removal rate of the other four pure strains was the highest (65%). Abo-Amer (2011) also observed that the pesticide degradation rate of the mixed bacteria reached 99% within 11 days, which was most likely due to the presence of different types of microorganisms in the mixed flora, such as bacterial archaea and fungi. There were some synergistic mechanisms among these bacteria to promote degradation.

**FIGURE 3:**
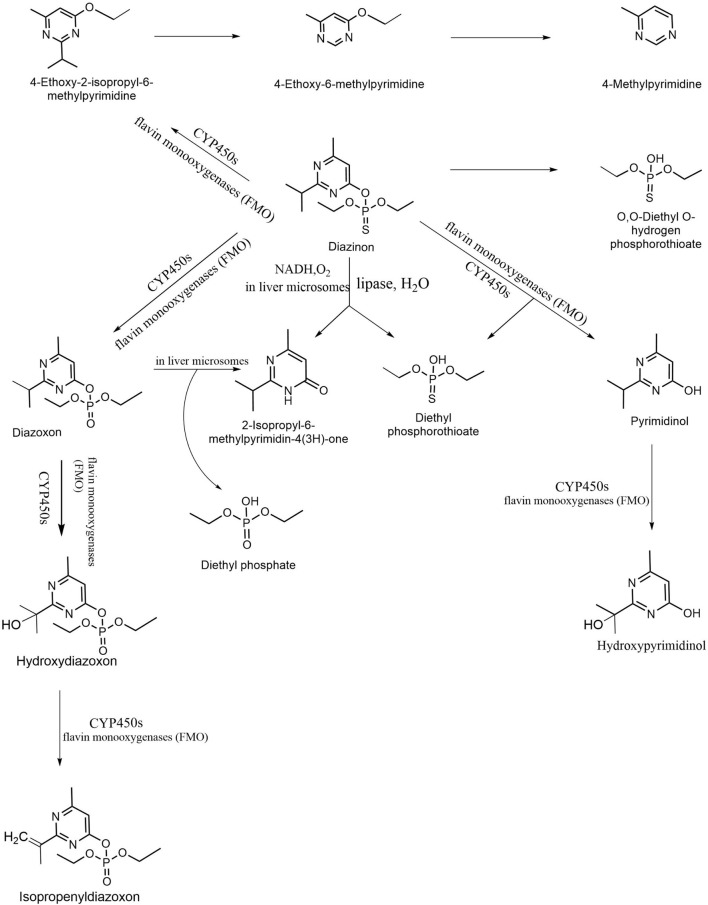
Proposed microbial degradation pathways of diazinon in microorganisms (Wang and Liu, 2016; Zhao et al., 2020).

In the process of pesticide degradation, hydrolysis is the main method (Kumar et al., 2018). The possible microbial degradation pathways of diazinon are shown in Figure 3. Various bacterial enzymes, such as acid and alkaline phosphatase, phosphodiesterase, phosphotriesterase (PTE), and dehydrogenase, have the ability to form hydrolytic functional groups in a short time. Combined microbial enzyme action can also achieve a detoxification effect (Briceño et al., 2018). The glutathione S-transferase superfamily is a key enzyme in biological metabolism. The enzyme bmGSTu2 exists in the silkworm *Bombyx mori*. It is a diazinon-metabolizing enzyme that can combine with 1-chloro-2,4-dinitrobenzene and contribute to the detoxification of diazine (Yamamoto and Yamada, 2016). Carboxylesterase is very effective in the detoxification of organophosphorus insecticides, and its mechanism is mainly divided into the following steps (Wheelock et al., 2008). It is first activated by a mixture of functional oxidases (MFOs) to form an active form of oxon. Second, organophosphorus insecticides combine with esterase and hydrolyze to release nitrophenol. Phosphorylated esterases may release phosphate groups to regain catalytic activity, or they may form phosphate complexes and lose catalytic activity (Wheelock et al., 2005). Both phosphorylase and methylcarbamoylase are helpful in reducing the toxicity of organophosphorus insecticides, but their stability is much higher than that of methylcarbamoylase (Casida and Quistad, 2004). Therefore, in terms of detoxification strength, phosphorylase works better.

## Molecular Mechanism of Diazinon Biodegradation

**FIGURE 4:**
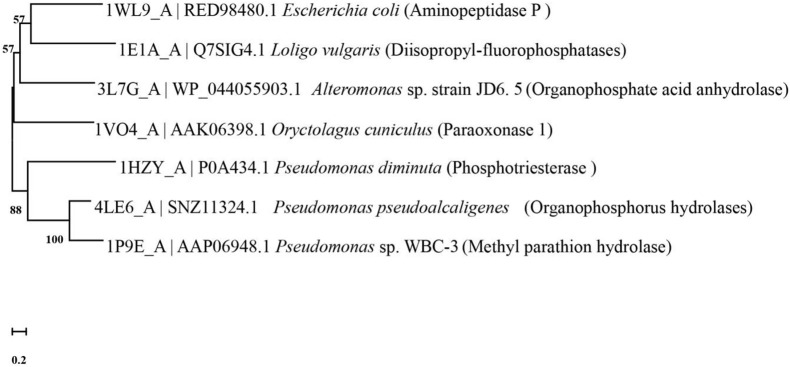
Phylogenetic tree of key diazinon-degrading enzymes constructed by the neighbor-joining method. The code before the strain name is the NCBI accession number. Aminopeptidase P was isolated from *Escherichia coli* (Graham et al., 2005). Diisopropyl-fluorophosphatase was isolated from *Loligo vulgaris* (Scharff et al., 2001). Organophosphate acid anhydrolase was isolated from *Alteromonas* sp. strain JD6.5 (Vyas et al., 2010). Paraoxonase 1 was isolated from *Oryctolagus cuniculus* (Thakur et al., 2019). Phosphotriesterase was isolated from *Pseudomonas diminuta* (Benning et al., 2001). Organophosphorus hydrolase was isolated from *Pseudomonas pseudoalcaligenes* (Gotthard et al., 2013). Methyl parathion hydrolase was isolated from *Pseudomonas* sp. WBC-3 (Dong et al., 2005).

Microbe-mediated bioremediation and catalysis have been confirmed by the previous literature, and a variety of strains have been used to degrade organophosphorus pesticides and have been found to achieve good remediation effects (Zhan et al., 2018a; Mishra et al., 2020; Huang et al., 2021). The root cause of this is that various strains contain a variety of enzymes that detoxify organophosphorus pesticides, most of which belong to phosphotriesterase (PTE) (da Silva et al., 2013; Birolli et al., 2019). Among them, organophosphorus hydrolase (OPH), methylparathion hydrolase (MPH), organophosphorus anhydrase (OPAA), diisopropyl-fluorophosphatase (DFPase) and paraoxonase 1 (PON1) are all classic degrading enzymes (da Silva et al., 2013; Daczkowski et al., 2015). These enzymes have their own characteristics. Figure 4 describes the evolutionary relationships between the functional enzymes involved in the degradation of diazinon.

To better study the degradation mechanism of enzymes, the most important step is to understand the enzyme itself. OPH is a zinc-containing homodimeric protein (Dumas et al., 1989). OPH carries the OPD gene of *Flavobacterium* sp. ATCC 27551 and *B. diminuta* MG. It uses Co^2+^, Zn^2+^, Mg^2+^, Ca^2+^, and Fe^2+^ for nucleophilic attack, thus hydrolyzing P-O, P-CN, P-F, and S bonds (Ghanem and Raushel, 2005; Orbulescu et al., 2006). MPH was isolated from *Plesiomonas* sp. M6 (M6-mph). Organophosphorus anhydrase (OPAA) is a dipeptidase isolated from *Monomonas*. With Mn^2+^ as the ligand, OPAA binds with the substrate to degrade organophosphorus by nucleophilic attack (Thakur et al., 2019). The degradation of DFP by the DFP enzyme may be due to its three histidine residues acting on the active sites of the substrate, of which two histidine residues, H274 and H174, can act as stabilizers, and H287 can achieve alkaline catalysis (Blum and Chen, 2010). The P-F bonds of the substrate are hydrolyzed gradually, eventually releasing isopropyl phosphate and fluoride (Jacquet et al., 2016). Paraoxonase 1 has the universality of a substrate, and it can degrade the oxon metabolites of parathion, diazinon, and chlorpyriphos (Draganov and La Du, 2004).

Diazinon belongs to the organophosphorus family and has a similar functional group structure. In the degradation of diazinon, each strain has its own degradation mechanism, and the enzyme interaction plays an important role in the catalytic degradation process (Chu et al., 2018; Vera et al., 2020; Zhao et al., 2020). These phosphotriester hydrolases adapt to a wide range of temperatures and pH values and are involved in the degradation of various OPS substrates. They have been reported to have great advantages in removing pesticides and nerve agents (Kapoor and Rajagopal, 2011; Gao et al., 2012; Lu et al., 2013). As an enzyme that catalyzes the stereoselective hydrolysis of a large number of triphosphate esters (Elias et al., 2008), triphosphoesterase (PTES, E.C. 3.1.8.1) can break the P-O, P-N or P-S bonds (Sogorb et al., 2004). Subsequently, microorganisms in nature use the hydrolysates of Ops as carbon/nitrogen sources (Kumar et al., 2018). Researchers have paid close attention to these hydrolases and proven their presence in microorganisms. By purifying, identifying and cloning-related genes, organophosphorus diminutases were isolated from *Brevundimonas* sp., *Pseudomonas diminuta*, and *Flavobacterium* sp. Paraoxonases are a class of interesting enzymes. According to sequence homology, PON enzymes can be divided into three groups: PON1, PON2, and PON3, among which PON1 has been the most studied for the degradation of diazinon (Draganov and La Du, 2004; Draganov et al., 2005). Paraoxonase 1 is a high-density lipoprotein-associated esterase/lactonase, which is also a monomer enzyme with calcium as the binding site. In the process of enzyme catalysis, it preferentially hydrolyzes the bonds of P-O, P-C, P-F, and P-CN.

The active site of an organophosphorus hydrolase contains one or two metal ions. Catalytic degradation of the substrate with metal ions is achieved through hydrogen bonding and the interaction of two amino acid residues in two active sites, followed by nucleophilic attack by hydroxide ions (Sethunathan and Yoshida, 1973). This also provides some basis for the catalytic triad previously mentioned. Organophosphorus hydrolases have similar active site geometries. The most typical PTE was detected in *Flavobacterium* sp. ATCC 27551 and *P. diminuta*, and the sequence homology of OpdA detected by *Astrobacterium radiobacter* was as high as 90% (Pedroso et al., 2014). Organophosphorus hydrolases have been confirmed by previous studies to have three amino acid residues to form a catalytic triad, of which the most common is hydroxy-serine residues that function as nucleophile attack substrates (Dar et al., 2019; Bhatt et al., 2021b).

**FIGURE 5:**
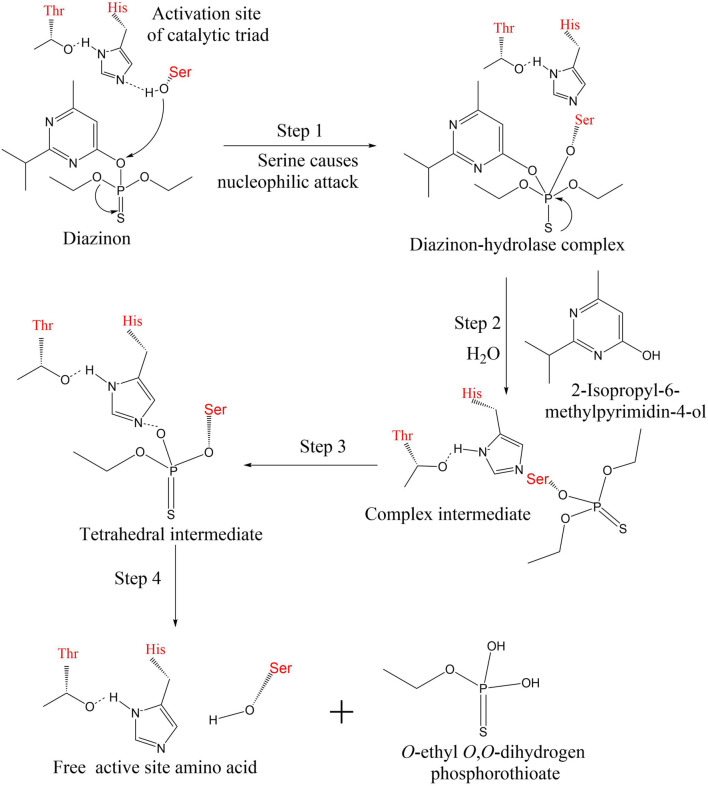
The specific enzymatic catalytic process of diazinon. The active site of organophosphorus hydrolase contains three amino acids (serine, histidine, threonine or glutamine) (Bhatt et al., 2020a; Huang et al., 2021). The hydroxyl group of serine takes part in nucleophilic reactions during diazinon biodegradation.

Three important amino acid residues (Ser-His-Thr or Glu) in PTE form the catalytic triad (Bhatt et al., 2020a). First, the substrate interacts with metal ions (hydrogen bonding) to activate hydroxy-serine residues. The reactive oxygen atoms on the serine residue nucleophilically attack the phosphorus atoms on diazinon, forming the diazinon–hydrolase complex (Islam et al., 2010). In the second step, hydroxide in the water molecule acts as a nucleophile, occupying the original active site of serine, while histidine acquires hydrogen protons (Huang et al., 2021). The remaining hydroxide continues to nucleophilically attack the phosphorus–oxygen bond, releasing the intermediate 2-isopropyl-6-methylpyrimidin-4-ol. During the third step, the serine activity decreased and returned to the resting state. A complex intermediate is present in the system (Zhan et al., 2020; Bhatt et al., 2021c). The oxygen atom in the phosphoric acid group is connected to the nitrogen atom in the base by a hydrogen bond, and the intermediate product is tetrahedral in configuration. In the last step, alcohols and free amino acids are separated from the complex through protonation, resulting in the detoxification of the toxic organophosphorus pesticide diazinon. The specific enzymatic catalytic process is shown in Figure 5.

The aryl dialkyl phosphatase (ADPB) isolated from *Nocardia* strain B-L is different from the former. Organic phosphate dehydrogenase is a dipeptidase isolated from both *Alteromonas undina* and *Alteromonas haloplankton*, and it has a relatively low hydrolysis rate (Cheng et al., 1993). In addition, methyl parathion hydrolase (MPH), isolated from *Plesiomonas* sp. M6 plays an important role in the hydrolysis of many OP_S_, including methyl parathion, chlorpyrifos, thiophos and diazinon (Cui et al., 2001; Liu et al., 2005). Chu et al. (2010) isolated another hydrolase from *Pseudomonas pseudoaligenes* strain C_2–1_. Interestingly, this enzyme was encoded by the *ophc*2 gene and had 46.4% similarity with the MPH gene. Sogorb and Vilanova (2002) believe that amylase from *Bacillus amyloliquefaciens* YP6 contains a variety of promising genes, including soluble phosphorus and OP_S_ degradation-related genes. In the process of microalgae degradation of OP_S_, it was observed that when the wavelength was 600 nm, the OD value increased linearly with time and the activity of carboxylesterase in microalgae increased, thus promoting OP_S_ to generate phosphate (Kumar et al., 2018).

The existence of a variety of hydrolase genes (*pho*D, *pho*A, *phr*C, *pho*E, *ycs*E, *bcr*C, and *yva*K) in microbial cells proves that microorganisms have potential advantages in agricultural environmental remediation, along with biosynthesis-related genes (*amh*X, *cge*E and *eps*M) and iron carrier biosynthesis-related genes (*ent*B, *men*F, *ent*C and *ent*A) (Sogorb and Vilanova, 2002; Idris et al., 2007; Chu et al., 2018). At present, we have found a variety of degrading enzymes and their related genes, but most of them describe the degradation of a single enzyme. Some individual enzymes were unstable and could not be developed into industrial strains. Therefore, increasing efforts are required to carry out genetic modification according to the characteristics of these enzymes to improve their activity and tolerance to ensure the efficient degradation of organic pollutants. The genetic engineering mechanism of the strain needs to be further explored.

## Conclusion and Future Perspectives

In recent years, diazinon has occupied an important position in the list of pesticides worldwide, and its high toxicity and high residue cannot be ignored. Currently, many physical and chemical methods have been applied to eliminate diazinon, but some challenges remain, such as high equipment cost, uncertainty regarding intermediates and incomplete mineralization. Therefore, an eco-friendly, economic, and feasible processing method is required for the sustainable degradation of diazinon. Microorganisms, including bacteria, fungi, and algae, are widely used in the degradation of diazinon. Biochemical and genetic research into diazinon-degrading microbes is necessary. The degradation ability of pure culture strains was always limited, while the degradation effect of microorganisms, including bacteria, fungi and algae, in a mixed culture was more efficient. In the future, the application of gene modification, mixed cultures of bacteria and immobilization technology will be a relatively popular research field, which has significance for the development of bioremediation strategies for diazinon-contaminated soil. On the one hand, immobilized enzyme technology will improve the stability of enzyme activity and expand the pH value and temperature range of the enzyme to adapt to better degradation of pollutants. On the other hand, we can construct transgenic vectors to transfer biodegradable genes into organisms that are easy to manipulate and stable. The degradation gene can be fully expressed to effectively remove pollutants. In addition, we can fully mobilize the synergistic or antagonistic effects in the mixed bacteria to achieve efficient degradation of organic pollutants. Furthermore, the development of recent sequencing techniques could add to and accelerate the prediction of the molecular-level mechanism involved in diazinon degradation.

## Author Contributions

SC conceived the presented idea. XW contributed to the writing and prepared the figures and tables. ZL, SP, JL, ZZ, PB, SM, and SC participated in revising the manuscript. All authors approved it for publication.

## Conflict of Interest

The authors declare that the research was conducted in the absence of any commercial or financial relationships that could be construed as a potential conflict of interest.

## Publisher’s Note

All claims expressed in this article are solely those of the authors and do not necessarily represent those of their affiliated organizations, or those of the publisher, the editors and the reviewers. Any product that may be evaluated in this article, or claim that may be made by its manufacturer, is not guaranteed or endorsed by the publisher.
